# Exploring the “Urban Advantage” in Access to Immunization Services: A Comparison of Zero-Dose Prevalence Between Rural, and Poor and Non-poor Urban Households Across 97 Low- and Middle-Income Countries

**DOI:** 10.1007/s11524-024-00859-7

**Published:** 2024-05-20

**Authors:** Thiago M. Santos, Bianca O. Cata-Preta, Andrea Wendt, Luisa Arroyave, Cauane Blumenberg, Tewodaj Mengistu, Daniel R. Hogan, Cesar G. Victora, Aluisio J. D. Barros

**Affiliations:** 1 International Center for Equity in Health, Federal University of Pelotas, Rua Deodoro 1160, Pelotas, RS 96020-220 Brazil; 2 Universidade Federal Do Paraná, Rua Padre Camargo, 280, Curitiba, PR 80060-240 Brazil; 3 Programa de Pós-Graduação Em Tecnologia Em Saúde, Pontifícia Universidade Católica Do Paraná, Rua Imaculada Conceição 1155, Curitiba, PR 80215-901 Brazil; 4 Gavi, the Vaccine Alliance, Chemin du Pommier 40, 1218 Geneva, Switzerland

**Keywords:** Vaccination, Poverty areas, Health inequities, Global health

## Abstract

Urban children are more likely to be vaccinated than rural children, but that advantage is not evenly distributed. Children living in poor urban areas face unique challenges, living far from health facilities and with lower-quality health services, which can impact their access to life-saving vaccines. Our goal was to compare the prevalence of zero-dose children in poor and non-poor urban and rural areas of low- and middle-income countries (LMICs). Zero-dose children were those who failed to receive any dose of a diphtheria-pertussis-tetanus (DPT) containing vaccine. We used data from nationally representative household surveys of 97 LMICs to investigate 201,283 children aged 12–23 months. The pooled prevalence of zero-dose children was 6.5% among the urban non-poor, 12.6% for the urban poor, and 14.7% for the rural areas. There were significant differences between these areas in 43 countries. In most of these countries, the non-poor urban children were at an advantage compared to the urban poor, who were still better off or similar to rural children. Our results emphasize the inequalities between urban and rural areas, but also within urban areas, highlighting the challenges faced by poor urban and rural children. Outreach programs and community interventions that can reach poor urban and rural communities—along with strengthening of current vaccination programs and services—are important steps to reduce inequalities and ensure that no child is left unvaccinated.

**Supplementary Information:**

The online version contains supplementary material available at 10.1007/s11524-024-00859-7.

## Introduction

Vaccination is one of the most cost-effective health interventions, with a massive impact on public health [1], and one of the few interventions capable of eradicating a disease [2]. Despite substantial progress in increasing immunization coverage worldwide in the last two decades, in 2022, over 14 million children worldwide were left without any dose of the diphtheria-pertussis-tetanus (DPT) vaccine [3]. Being unvaccinated with DPT is used as a marker for children who have not received any routine vaccines, also called “zero-dose children” [3].

The World Health Organization (WHO) Immunization Agenda 2030 (IA2030) aims to achieve immunization for all by 2030, reducing the number of zero-dose children by 50% from its level in 2019 [2]. Around a quarter of zero-dose children live in urban and peri-urban areas, and another 11% in remote rural areas [4]. Altogether, nearly half of the zero-dose children live in deprived communities clustered in remote rural settings, areas affected by conflict, and disadvantaged urban settings [4–6].

Compared to people living in urban areas, those in rural areas often present lower coverage of reproductive, maternal, newborn, and child health interventions, such as antenatal care and child immunization [7, 8]. This is partly because, on average, urban households earn higher incomes, benefit from improved infrastructure, have better education, and reside closer to services. However, this urban advantage is not evenly distributed across urban households [9].

Urban populations have experienced significant growth in recent decades along with rapid and largely unplanned urbanization, particularly in LMICs, leading to over one billion people living in informal urban settlements or slums [10]. These people are most likely living in conditions that are detrimental to their health and well-being [11].

While few studies separate the poorest and more deprived urban dwellers from the urban population as a whole [12], important differences are often present. Poor and marginalized urban populations are vulnerable not only because of poverty but also due to low education, limited social support, poorly constructed houses, inadequate access to basic amenities, and a higher risk of exposure to natural disasters such as floods compared to rural and non-poor urban populations [13, 14]. They often live far from health facilities, access lower-quality health services, and face discrimination from healthcare staff [15]. Additionally, the COVID-19 pandemic and its associated economic crisis caused job losses and deprivation, hitting harder the already poor and vulnerable people. This group is called the “new poor” and is most likely found in cities [16].

Therefore, the so-called urban advantage is not for all urban dwellers and may mask severe inequities within urban areas. Previous studies acknowledged that rapid urbanization rates have resulted in large contingents of poor urban families, often presenting worse health indicators than rural residents [17, 18]. For instance, the urban neonatal mortality rate (38 per 1000 live births) was higher than the rural (20 per 1000 live births) in Tanzania; and the under-five mortality rate was much higher among the urban poor (73%) compared to the urban non-poor (42%) in India, but similar to the under-five mortality rate in rural areas (74%) [19, 20]. These findings point to possible challenges that may be unique to urban areas and the urban poor in particular and suggest the need for differentiated strategies to reach these populations with health services. This paper explores the prevalence of zero-dose children in 97 LMICs using national health surveys carried out since 2010, focusing on the differences between the urban non-poor, the urban poor, and rural children.

The study highlights the multidimensional aspects of geographic inequities in immunization coverage, which helps inform programmatic priorities and strategies to identify and reach zero-dose children. This is particularly important since urbanization is increasing across LMICs, with more than 60% of the world’s population expected to live in urban areas by 2030 [10].

## Methods

### Data sources and study sample

We used data from Demographic and Health Surveys (DHS) and Multiple Indicator Cluster Surveys (MICS) with information on DPT immunization. Both DHS and MICS are surveys conducted in LMICs with standardized questionnaires and similar sampling strategies [21]. We selected countries with at least one survey conducted since 2010, using the latest survey if more than one was available.

Our study sample comprised children 12–23 months of age, the usual age range used for immunization indicators. The exceptions—due to measles vaccination being offered in the second year of life—were Egypt (2004), Bosnia and Herzegovina (2011), Jamaica (2011), Ukraine (2012), and Peru (2021), for which we studied children aged 18–29 months, and Türkiye (2013), for which we studied children aged 15–26 months. Our outcome does not include measles vaccination, but we chose this approach to make our coverage indicator consistent with results from the published survey reports [22, 23].

### Zero-dose indicator

Zero dose was defined as children in the study age range who failed to receive any dose of a DPT-containing vaccine. We used zero-DPT as a proxy for zero-dose children to be coherent with the IA2030 definition of zero-dose [2].

DPT vaccination data were collected from home-based child vaccination records or maternal report if information was missing in the vaccination records or no vaccination record was available. We treated children with missing information as not vaccinated according to WHO recommendations [24].

### Wealth and area of residence classification

We classified the sampled households into rural, poor urban, and non-poor urban. Rural and urban households were defined according to the countries’ classification of residence area, regardless of their wealth. Urban households were further divided into poor and non-poor.

Each DHS and MICS survey includes a household wealth score based on the ownership of assets, building materials, electricity availability, type of water supply, and sanitary facilities, among other variables [25]. Most wealth-related variables are common to urban and rural settings, but there are specific ones, especially for rural households. We used the urban-specific wealth score—based on principal components analysis of the relevant urban variables—to classify the urban population into poor and non-poor. The households with the 40% lowest wealth scores were classified as poor, and the remaining 60% as non-poor, weighted by household size.

### Statistical analysis

We calculated each country’s zero-dose prevalence and 95% confidence interval (95% CI) separately. In all analyses, estimates considered the sample design, including clustering, weights, and strata. We presented the distribution of rural, poor urban, and non-poor urban children and the zero-dose prevalence for each group.

We calculated zero-dose prevalence ratios comparing rural to urban-poor children. We do not present the result for Maldives, Serbia, Tonga, and Rwanda because the zero-dose prevalence for urban-poor children was nil.

In the figures, countries are grouped by UNICEF world regions: West and Central Africa (WCA), Eastern and Southern Africa (ESA), Middle East and North Africa (MENA), Eastern Europe and Central Asia (EECA), South Asia (SA), East Asia and Pacific (EAP), and Latin America and the Caribbean (LAC). We also present pooled estimates at the global level. They were calculated using Poisson regression controlling for country, to avoid ecological bias. Pooled results were weighted by the national population of children aged 12–23 months in each country.

All analyses were conducted using R (version 4.3.1, R Foundation for Statistical Computing, Vienna, Austria) and Stata (StataCorp. 2021. Stata Statistical Software: Release 17. College Station, TX: StataCorp LLC.).

## Results

Our analyses included 97 LMICs and 201,283 children. The pooled weighted prevalence of zero-dose children was 12.6% (95% CI 12.2–13.0%) for all children, 9.1% (8.6–9.6%) for urban children, and 14.7% (14.2–15.2%) for rural children. Supplementary Table 1 lists the countries included in the study and their national zero-dose prevalence.

**Fig. 1:**
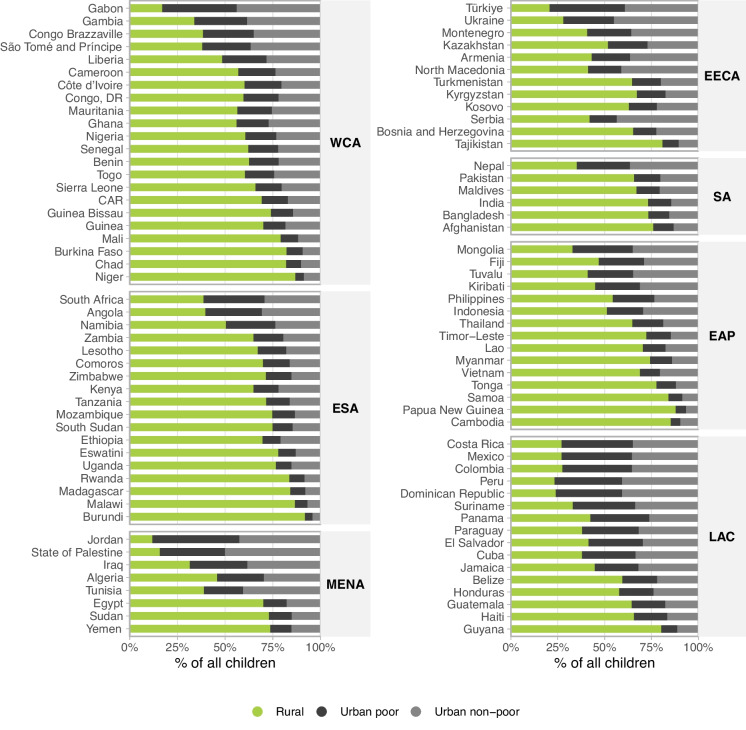
Distribution of children living in rural, poor urban, and non-poor urban areas across 97 countries. Legend: WCA, West and Central Africa; ESA, Eastern and Southern Africa; MENA, Middle East and North Africa; EECA, Eastern Europe and Central Asia; SA, South Asia; EAP, East Asia and Pacific; LAC, Latin America and Caribbean

Figure 1 illustrates the distribution of children living in rural and poor and non-poor urban households in each country. Note that the proportion of poor households among the urban ones is fixed at 40%. The proportion of rural children ranged from 11.8% in Jordan (MENA) to 91.8% in Burundi (ESA), with a median of 51.8%. The proportion of poor urban children among all children in each country ranged from 3.9% in Burundi to 45.6% in Jordan, with a median of 24.7%. Non-poor urban children ranged from 4.3% in Burundi to 50.2% in the State of Palestine, with a median of 27.3% (Fig. 1 and Supplementary Table 2).

**Fig. 2:**
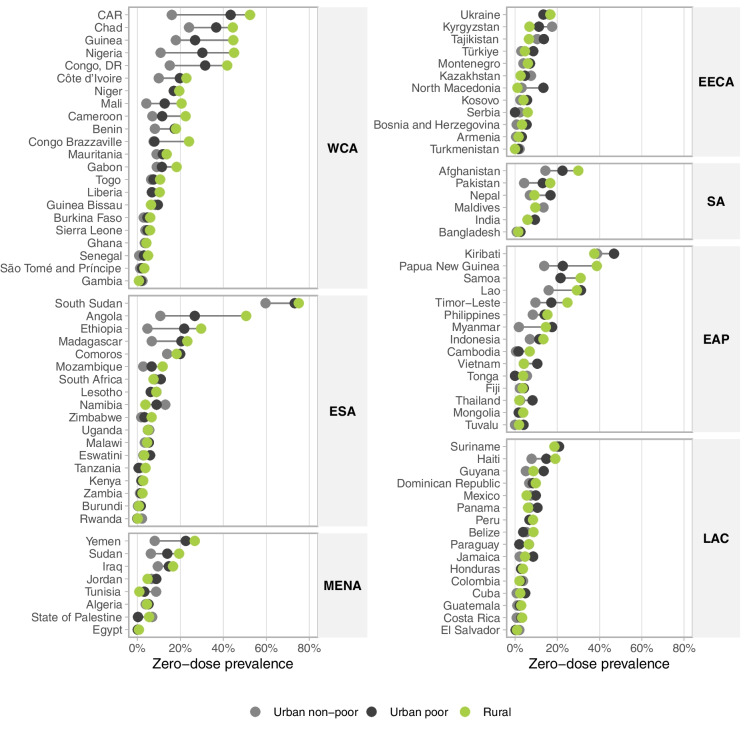
Zero-dose prevalence stratified by non-poor urban, poor urban, and rural households. Legend: WCA, West and Central Africa; ESA, Eastern and Southern Africa; MENA, Middle East and North Africa; EECA, Eastern Europe and Central Asia; SA, South Asia; EAP, East Asia and Pacific; LAC, Latin America and Caribbean

Figure 2 shows the zero-dose prevalence for the three study groups for each country. The pooled weighted prevalence of zero-dose children was 6.5% (95% CI 6.0–7.0%) among non-poor urban children, 12.6% (11.7–13.5%) for poor urban children, and 14.7% (14.2–15.2%) for rural children. At the country level, in most cases with a noticeable difference, the highest zero dose prevalence was observed in rural children, followed by the urban poor. Especially in countries with low zero-dose prevalence, zero-dose levels were comparable among rural, poor urban, and non-poor urban children. There were significant between-group differences (*p* < 0.05) in 43 countries, representing 44% of those studied (Supplementary Table 2). The median zero-dose prevalence in these countries was 7.8% (interquartile range: 4.8–10.9%). Among the urban poor, zero-dose prevalence was either between the prevalence observed in rural and non-poor urban children or similar to that of rural children.

The widest gaps between groups were found in WCA (median zero-dose prevalence of 13.1%) and ESA (median of 6.4%). The Central African Republic (CAR), Chad, Guinea, Nigeria, the Democratic Republic of Congo, and Angola presented the greatest absolute inequalities. The smallest absolute inequalities were found in the LAC region, where the median prevalence was 6.1%. Supplementary Table 2 presents zero-dose prevalence among rural, poor urban, and non-poor urban children in each country.

**Fig. 3:**
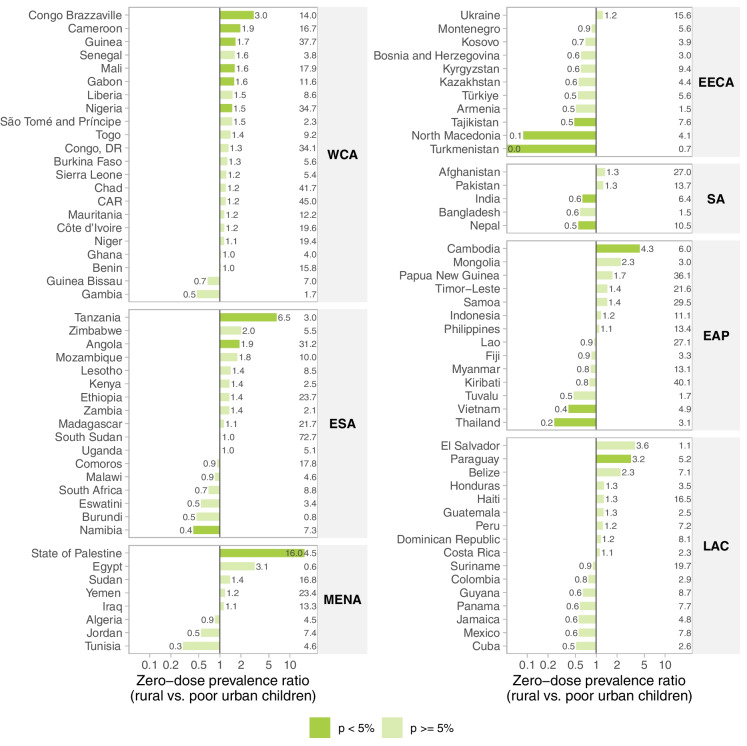
Zero-dose prevalence ratio between rural and poor urban children. Legend: WCA, West and Central Africa; ESA, Eastern and Southern Africa; MENA, Middle East and North Africa; EECA, Eastern Europe and Central Asia; SA, South Asia; EAP, East Asia and Pacific; LAC, Latin America and Caribbean. Note: The numbers closest to the bars are the zero-dose prevalence ratios (rural vs. poor urban children). The numbers to the right of each panel are the national zero-dose prevalence

Figure 3 compares zero-dose prevalence between rural and poor urban children using the ratio between rural and poor urban children. Across multiple countries, zero-dose prevalence tended to be higher among rural children compared to the urban poor, although most of these differences did not reach statistical significance. Notably, in 11 countries (11%), rural children exhibited a significantly higher zero-dose prevalence, whereas in eight countries (8%), the reverse trend was observed. The 11 countries where the prevalence was higher among rural children were Angola, Congo Brazzaville, Cambodia, Cameroon, Guinea, Mali, Gabon, Nigeria, Paraguay, State of Palestine, and Tanzania. The eight countries with higher prevalence among the urban poor were India, Namibia, Nepal, North Macedonia, Tajikistan, Thailand, Turkmenistan, and Vietnam.

In summary, zero-dose prevalence was lower for non-poor urban children than for poor urban and rural children. Poor urban and rural children had similar prevalence, albeit slightly lower for the urban poor. Countries with the largest absolute inequalities were found in WCA and ESA, while LAC had the smallest.

## Discussion

We evaluated the prevalence of zero-dose children in poor urban, non-poor urban, and rural households in 97 LMICs. The countries included in the analyses represent 76% of all low-income, 81% of lower-middle-income, and 59% of upper-middle-income countries.

Our analyses revealed that zero-dose prevalence differs between these groups in 43% of the countries studied. Poor urban children were more likely to be zero-dose than rural children in only eight countries, while non-poor urban children were systematically better off than rural and poor urban children in most countries where a difference was observed.

Overall, the literature assessing urban–rural disparities in childhood immunization in LMICs shows rural children to be worse off regarding timely vaccination and coverage in most countries [26, 27]. However, rapid urbanization often results in informal urban settlements where people live in poor and overcrowded households lacking basic infrastructure. On the other hand, poor urban children tend to live closer to health facilities than rural children, which may offset socioeconomic disadvantage. How the health systems work in each country will be an important aspect of how much this disadvantage is offset. Comparisons of how poor urban children fare compared to non-poor urban and rural children in terms of health intervention coverage are therefore relevant when trying to minimize inequities in vaccination.

Few studies have compared the health indicators of mothers and children in poor urban families to those living in rural areas. The available studies provide contradictory results, sometimes indicating the urban advantage and sometimes indicating better outcomes in the rural population [18, 28, 29]. The direction of this effect is highly context dependent. One study found that living in slum areas was associated with higher child mortality in LMICs, but the effect was attenuated with adjustment for access to antenatal care [30], suggesting that better-structured health systems can reduce socioeconomic disadvantage on health. In sub-Saharan African countries, higher urban population growth rates were inversely associated with immunization coverage among urban children [28].

Our analyses showed that the differences between the three groups of children were generally small in countries with zero-dose prevalence below 10%. Few of these countries presented significant differences, and Cambodia and Namibia are two examples with opposite patterns. In Cambodia, the zero-dose prevalence is less than 1% among the urban non-poor compared to 7% among rural children. In Namibia, 13% of non-poor urban children were zero-dose compared to 4% of rural children. Even though the zero-dose prevalence in these groups may not be immediately alarming, the gaps between urban and rural children were large, and the existence of zero-dose children is unacceptable.

The widest gaps in zero-dose were observed for countries with higher prevalence, where we systematically see an urban advantage and rural children being in the worst situation. This is the case of countries like CAR and Chad from WCA, South Sudan and Angola in ESA, Yemen in MENA, and Papua New Guinea in EAP. Countries in this situation need an overall improvement of their immunization services, which can leave 50% or more of children without a single dose of vaccine. Distance to health facilities, marginalization, and multiple deprivation must be tackled, along with offering a reliable supply of vaccines to avoid the frustration of stockouts for children after a long journey to a facility [16].

In 11% of countries, the prevalence ratio of zero-dose showed a higher prevalence of zero-dose children among rural compared to poor urban children. All of these were low- or lower-middle-income countries, except for Angola, Gabon, and Paraguay. Considering only the African continent, 17% of countries presented this pattern, which may be explained by several barriers rural people face in accessing health care services such as workforce shortages, transportation issues, and distance from health facilities [31]. It is worth noting that the assessment of ratios does not always match the visual impression of absolute gaps offered by the equiplots in Fig. 2. Ratios increase rapidly when the denominator is small, making, for instance, the situation of the State of Palestine look extreme, while the zero-dose prevalence in rural children, at 5.6%, is relatively low compared to other countries. The reason is that the prevalence among the urban poor is 0.3%, yielding a ratio of 16.0 (based on the non-rounded proportions).

Among the countries where zero-dose prevalence was higher among poor urban children than in rural children (8% of all countries), all were upper-middle income countries, except for Nepal, classified as lower-middle income. In these countries, it is possible that the disadvantages of urban slums are more strongly affecting the poor urban populations than in other settings.

Our results highlight both the challenges and the opportunities in achieving simultaneously the Sustainable Development Goals 3 (“Ensure healthy lives and promote well-being for all at all ages,” including universal access to vaccines [32]) and 11 (“Make cities and human settlements inclusive, safe, resilient and sustainable” [33]) in the context of LMICs. The urban poor are often more mobile, have more inflexible work schedules, and are from diverse cultural backgrounds [34]. This makes them harder to reach by health workers and standardized public health interventions and programs, increasing the barriers to continuity of care [34]. Interventions with community involvement that tackle the specific needs of poor urban communities are a possible pathway for reducing those inequalities and the number of zero-dose children [35]. They can include flexible and extended service hours, staff training, geographical monitoring, outreach programs, new vaccination sites (e.g., stadiums, markets, and malls), community support groups and mobilization (including traditional and religious leaders), text message reminders, and even financial incentives [34–36].

Our findings showed that, in most cases where there are considerable differences, the non-poor urban children are at an advantage compared to the urban poor, who are still better off than rural children. Most likely, children in urban settings end up having easier access to health services, despite their level of deprivation. However, our categorization of urban children may not be sensitive enough to single out the most deprived or those living in the worst conditions. Also, the most affected households in terms of lack of health access and infrastructure may be limited to slum areas in large cities or metropolitan areas.

Identifying the urban poor, urban slums, or informal settlements is challenging, particularly in population surveys. The United Nations Habitat defines a slum household based on characteristics such as access to clean water, improved sanitation, adequate living space, and secure tenure [11]. Another approach, proposed by Fink et al., is based on the cluster where the household is and defines an informal neighborhood if 75% of households lack a minimal housing structure [29]. These approaches are interesting but challenging to apply to household surveys as they are not representative of small areas.

Therefore, we relied on the wealth index provided with the DHS and MICS survey datasets to classify poor and non-poor urban households, using a cut-off point of 40% poorest that is in line with several papers that dichotomized households into poor and non-poor [37–39]. This index is known to underestimate the wealth of rural households, but in our case, it was used to classify only urban households, minimizing any possible bias. Nevertheless, our approach relies solely on the household characteristics, failing to take into account the neighborhood in which the household is located and the detrimental effects of being surrounded by poor sanitation infrastructure and unsafe environments [29]. Furthermore, neighborhoods in large cities are often heterogenous, and wealthier households can be surrounded by poorer neighbors and vice versa [40].

Another limitation is that home-based vaccination records were unavailable for approximately one-quarter of the children, and information for them was obtained by caregiver recall. However, given that we are using a zero-dose indicator, the potential of bias is reduced.

DHS and MICS are highly comparable surveys [21], using almost identical immunization modules [22, 23]. One key difference between them is the fact that MICS includes orphans and foster children in the vaccination module, while DHS does not [21]. This is due to DHS collecting vaccination information only from biological mothers, while MICS also includes other primary caregivers [21]. Nevertheless, for undernutrition, the inclusion or exclusion of orphans and foster children did not substantially impact national estimates [21].

## Conclusions

Our results show a considerable number of countries with a sizeable prevalence of zero dose. These countries also presented a systematic disadvantage for poor urban and rural children compared to non-poor urban children. Context-specific situations must be considered in strengthening policies and programs so that they can reach all children with vaccines.

### Supplementary Information

Below is the link to the electronic supplementary material.

Supplementary file1 (DOCX 71 KB)

## Data Availability

All the analyses were carried out using publicly available datasets that can be obtained directly from the DHS (dhsprogram.com) the MICS (mics.unicef.org) websites.
