# Supplementary material for: Exploring the “Urban Advantage” in Access to Immunization Services: A Comparison of Zero-Dose Prevalence Between Rural, and Poor and Non-poor Urban Households Across 97 Low- and Middle-Income Countries
Source: J Urban Health. 2024 May 20;101(3):638–47. doi: 10.1007/s11524-024-00859-7 (PMC11189869; doi:10.1007/s11524-024-00859-7)
Supplement: Supplementary file 1 — Supplementary file1 (DOCX 71 KB) [file 11524_2024_859_MOESM1_ESM.docx]

Supplementary material

Supplementary Table 1 – Countries included and national zero-dose prevalence.

| Country | Survey year | Source | Zero-dose (%) | 95% CI lower limit | 95% CI upper limit |
| --- | --- | --- | --- | --- | --- |
| Afghanistan | 2015 | DHS | 27.0 | 23.8 | 30.5 |
| Angola | 2015 | DHS | 31.2 | 28.6 | 34.0 |
| Armenia | 2015 | DHS | 1.5 | 0.7 | 3.3 |
| Burundi | 2016 | DHS | 0.8 | 0.5 | 1.2 |
| Benin | 2017 | DHS | 15.8 | 13.8 | 17.9 |
| Burkina Faso | 2010 | DHS | 5.6 | 4.5 | 7.1 |
| Bangladesh | 2017 | DHS | 1.5 | 0.9 | 2.5 |
| Bosnia and Herzegovina | 2011 | MICS | 3.0 | 1.8 | 4.9 |
| Belize | 2015 | MICS | 7.1 | 4.4 | 11.2 |
| Central African Republic | 2018 | MICS | 45.0 | 41.7 | 48.3 |
| Cote d'Ivoire | 2016 | MICS | 19.6 | 17.3 | 22.2 |
| Cameroon | 2018 | DHS | 16.7 | 14.2 | 19.5 |
| Congo, DR | 2017 | MICS | 34.1 | 30.2 | 38.2 |
| Congo Brazzaville | 2014 | MICS | 14.0 | 12.0 | 16.3 |
| Colombia | 2010 | DHS | 2.9 | 2.2 | 3.8 |
| Comoros | 2012 | DHS | 17.8 | 14.1 | 22.2 |
| Costa Rica | 2018 | MICS | 2.3 | 1.1 | 4.7 |
| Cuba | 2019 | MICS | 2.6 | 1.3 | 5.0 |
| Dominican Republic | 2019 | MICS | 8.1 | 6.5 | 9.9 |
| Algeria | 2018 | MICS | 4.5 | 3.7 | 5.5 |
| Egypt | 2014 | DHS | 0.6 | 0.3 | 1.1 |
| Ethiopia | 2019 | DHS | 23.7 | 19.2 | 28.9 |
| Fiji | 2021 | MICS | 3.3 | 1.9 | 5.7 |
| Gabon | 2012 | DHS | 11.6 | 9.0 | 14.7 |
| Ghana | 2017 | MICS | 4.0 | 2.8 | 5.8 |
| Guinea | 2018 | DHS | 37.7 | 34.1 | 41.4 |
| Gambia | 2019 | DHS | 1.7 | 0.9 | 3.0 |
| Guinea Bissau | 2018 | MICS | 7.0 | 5.2 | 9.4 |
| Guatemala | 2014 | DHS | 2.5 | 1.8 | 3.3 |
| Guyana | 2019 | MICS | 8.7 | 6.2 | 12.2 |
| Honduras | 2019 | MICS | 3.5 | 2.5 | 4.8 |
| Haiti | 2016 | DHS | 16.5 | 13.3 | 20.1 |
| Indonesia | 2017 | DHS | 11.1 | 9.8 | 12.7 |
| India | 2019 | DHS | 6.4 | 6.0 | 6.7 |
| Iraq | 2018 | MICS | 13.3 | 11.4 | 15.5 |
| Jamaica | 2011 | MICS | 4.8 | 2.5 | 9.3 |
| Jordan | 2017 | DHS | 7.4 | 5.7 | 9.7 |
| Kazakhstan | 2015 | MICS | 4.4 | 3.2 | 6.0 |
| Kenya | 2014 | DHS | 2.5 | 1.9 | 3.3 |
| Kyrgyzstan | 2018 | MICS | 9.4 | 6.7 | 12.9 |
| Cambodia | 2014 | DHS | 6.0 | 4.6 | 7.9 |
| Kiribati | 2018 | MICS | 40.1 | 35.1 | 45.4 |
| Laos | 2017 | MICS | 27.1 | 24.7 | 29.7 |
| Liberia | 2019 | DHS | 8.6 | 6.3 | 11.5 |
| Lesotho | 2018 | MICS | 8.5 | 6.3 | 11.2 |
| Madagascar | 2021 | DHS | 21.7 | 19.4 | 24.3 |
| Maldives | 2016 | DHS | 9.2 | 6.6 | 12.8 |
| Mexico | 2015 | MICS | 7.8 | 5.6 | 10.7 |
| North Macedonia | 2018 | MICS | 4.1 | 1.7 | 9.5 |
| Mali | 2018 | DHS | 17.9 | 15.1 | 21.1 |
| Myanmar | 2015 | DHS | 13.1 | 10.1 | 16.9 |
| Montenegro | 2013 | MICS | 5.6 | 2.7 | 11.0 |
| Mongolia | 2018 | MICS | 3.0 | 1.9 | 4.5 |
| Mozambique | 2015 | DHS | 10.0 | 6.8 | 14.4 |
| Mauritania | 2019 | DHS | 12.2 | 10.1 | 14.6 |
| Malawi | 2019 | MICS | 4.6 | 3.7 | 5.7 |
| Namibia | 2013 | DHS | 7.3 | 5.5 | 9.7 |
| Niger | 2021 | DHS | 19.4 | 15.8 | 23.6 |
| Nigeria | 2018 | DHS | 34.7 | 32.7 | 36.7 |
| Nepal | 2019 | MICS | 10.5 | 8.5 | 13.0 |
| Pakistan | 2017 | DHS | 13.7 | 11.2 | 16.5 |
| Panama | 2013 | MICS | 7.7 | 5.5 | 10.8 |
| Peru | 2021 | DHS | 7.2 | 6.3 | 8.4 |
| Philippines | 2017 | DHS | 13.4 | 11.5 | 15.5 |
| Papua New Guinea | 2016 | DHS | 36.1 | 32.2 | 40.2 |
| Paraguay | 2016 | MICS | 5.2 | 3.9 | 6.9 |
| State of Palestine | 2019 | MICS | 4.5 | 3.5 | 5.8 |
| Rwanda | 2019 | DHS | 0.4 | 0.2 | 0.9 |
| Sudan | 2014 | MICS | 16.8 | 14.3 | 19.6 |
| Senegal | 2019 | DHS | 3.8 | 2.6 | 5.6 |
| Sierra Leone | 2019 | DHS | 5.4 | 4.2 | 6.9 |
| El Salvador | 2014 | MICS | 1.1 | 0.5 | 2.5 |
| Serbia | 2019 | MICS | 3.5 | 1.8 | 6.7 |
| South Sudan | 2010 | MICS | 72.7 | 69.6 | 75.5 |
| Sao Tome and Principe | 2019 | MICS | 2.3 | 1.1 | 4.7 |
| Suriname | 2018 | MICS | 19.7 | 15.5 | 24.7 |
| Eswatini | 2014 | MICS | 3.4 | 2.1 | 5.6 |
| Chad | 2014 | DHS | 41.7 | 38.7 | 44.8 |
| Togo | 2017 | MICS | 9.2 | 6.8 | 12.2 |
| Thailand | 2019 | MICS | 3.1 | 1.8 | 5.4 |
| Tajikistan | 2017 | DHS | 7.6 | 6.1 | 9.4 |
| Turkmenistan | 2015 | MICS | 0.7 | 0.3 | 1.5 |
| Timor Leste | 2016 | DHS | 21.6 | 18.8 | 24.7 |
| Tonga | 2019 | MICS | 3.6 | 1.4 | 8.8 |
| Tunisia | 2018 | MICS | 4.6 | 3.1 | 6.7 |
| Türkiye | 2013 | DHS | 5.6 | 3.7 | 8.3 |
| Tuvalu | 2019 | MICS | 1.7 | 0.4 | 7.2 |
| Tanzania | 2015 | DHS | 3.0 | 2.2 | 4.2 |
| Uganda | 2016 | DHS | 5.1 | 4.1 | 6.3 |
| Ukraine | 2012 | MICS | 15.6 | 12.1 | 20.0 |
| Vietnam | 2020 | MICS | 4.9 | 3.3 | 7.1 |
| Samoa | 2019 | MICS | 29.5 | 25.0 | 34.5 |
| Kosovo | 2019 | MICS | 3.9 | 2.2 | 7.0 |
| Yemen | 2013 | DHS | 23.4 | 21.2 | 25.8 |
| South Africa | 2016 | DHS | 8.8 | 6.4 | 12.2 |
| Zambia | 2018 | DHS | 2.1 | 1.4 | 3.1 |
| Zimbabwe | 2019 | MICS | 5.5 | 3.9 | 7.6 |

Note: zero-dose is defined as children who failed to receive any doses of DPT-containing vaccine.

Supplementary Table 2 – Zero-dose prevalence in urban poor, urban non-poor and rural children for each country included.

| Country | Group | Proportion of children (%) | Zero-dose (%) | 95% CI lower limit | 95% CI  upper limit | p value* |
| --- | --- | --- | --- | --- | --- | --- |
| Afghanistan | urban non-poor | 13.2 | 14.4 | 10.6 | 19.2 | **< 0.01** |
| Afghanistan | urban poor | 10.9 | 22.4 | 16.8 | 29.2 |  |
| Afghanistan | rural | 75.9 | 29.9 | 25.8 | 34.4 |  |
| Algeria | urban non-poor | 29.8 | 4.0 | 2.7 | 5.9 | 0.559 |
| Algeria | urban poor | 24.6 | 5.2 | 3.7 | 7.3 |  |
| Algeria | rural | 45.7 | 4.5 | 3.2 | 6.2 |  |
| Angola | urban non-poor | 30.8 | 10.7 | 7.6 | 14.8 | **< 0.01** |
| Angola | urban poor | 29.6 | 26.7 | 22.5 | 31.4 |  |
| Angola | rural | 39.6 | 50.6 | 45.8 | 55.5 |  |
| Armenia | urban non-poor | 36.5 | 0.5 | 0.1 | 3.4 | 0.226 |
| Armenia | urban poor | 20.5 | 3.2 | 1.2 | 8.4 |  |
| Armenia | rural | 43.0 | 1.6 | 0.4 | 6.0 |  |
| Bangladesh | urban non-poor | 15.6 | 0.8 | 0.2 | 3.7 | 0.387 |
| Bangladesh | urban poor | 11.2 | 2.6 | 1.0 | 6.4 |  |
| Bangladesh | rural | 73.2 | 1.5 | 0.8 | 2.8 |  |
| Belize | urban non-poor | 22.1 | 5.3 | 1.6 | 16.7 | 0.349 |
| Belize | urban poor | 18.6 | 3.9 | 1.5 | 9.4 |  |
| Belize | rural | 59.3 | 8.7 | 4.7 | 15.6 |  |
| Benin | urban non-poor | 22.0 | 8.2 | 5.9 | 11.2 | **< 0.01** |
| Benin | urban poor | 15.6 | 17.4 | 12.2 | 24.2 |  |
| Benin | rural | 62.5 | 18.0 | 15.5 | 20.8 |  |
| Bosnia and Herzegovina | urban non-poor | 22.5 | 0.8 | 0.1 | 5.4 | 0.100 |
| Bosnia and Herzegovina | urban poor | 12.4 | 5.5 | 1.7 | 16.5 |  |
| Bosnia and Herzegovina | rural | 65.1 | 3.3 | 1.8 | 5.9 |  |
| Burkina Faso | urban non-poor | 9.5 | 3.0 | 1.2 | 7.4 | 0.156 |
| Burkina Faso | urban poor | 8.5 | 4.7 | 2.5 | 8.8 |  |
| Burkina Faso | rural | 82.0 | 6.0 | 4.7 | 7.7 |  |
| Burundi | urban non-poor | 4.3 | 0.3 | 0.0 | 2.2 | 0.297 |
| Burundi | urban poor | 3.9 | 1.7 | 0.4 | 6.6 |  |
| Burundi | rural | 91.8 | 0.8 | 0.5 | 1.3 |  |
| CAR | urban non-poor | 17.2 | 16.1 | 12.6 | 20.3 | **< 0.01** |
| CAR | urban poor | 13.8 | 43.4 | 34.3 | 53.1 |  |
| CAR | rural | 69.1 | 52.5 | 48.0 | 57.0 |  |
| Cambodia | urban non-poor | 9.7 | 0.5 | 0.1 | 2.7 | **< 0.01** |
| Cambodia | urban poor | 5.1 | 1.6 | 0.7 | 3.6 |  |
| Cambodia | rural | 85.2 | 6.9 | 5.3 | 9.1 |  |
| Cameroon | urban non-poor | 23.7 | 7.0 | 4.7 | 10.3 | **< 0.01** |
| Cameroon | urban poor | 19.4 | 11.6 | 7.9 | 16.5 |  |
| Cameroon | rural | 56.8 | 22.5 | 18.6 | 27.0 |  |
| Chad | urban non-poor | 10.3 | 24.0 | 18.0 | 31.3 | **< 0.01** |
| Chad | urban poor | 7.8 | 36.7 | 27.5 | 47.0 |  |
| Chad | rural | 81.9 | 44.4 | 40.9 | 48.0 |  |
| Colombia | urban non-poor | 35.5 | 3.8 | 2.5 | 5.8 | 0.233 |
| Colombia | urban poor | 37.2 | 2.7 | 1.8 | 4.1 |  |
| Colombia | rural | 27.4 | 2.1 | 1.2 | 3.6 |  |
| Comoros | urban non-poor | 16.3 | 13.9 | 8.4 | 22.1 | 0.482 |
| Comoros | urban poor | 14.0 | 20.0 | 12.0 | 31.5 |  |
| Comoros | rural | 69.7 | 18.3 | 13.6 | 24.1 |  |
| Congo Brazzaville | urban non-poor | 35.1 | 7.6 | 5.0 | 11.4 | **< 0.01** |
| Congo Brazzaville | urban poor | 26.6 | 8.0 | 5.2 | 12.0 |  |
| Congo Brazzaville | rural | 38.3 | 24.1 | 20.6 | 27.9 |  |
| Congo Democratic Republic | urban non-poor | 22.2 | 15.1 | 11.0 | 20.5 | **< 0.01** |
| Congo Democratic Republic | urban poor | 18.3 | 31.6 | 23.2 | 41.5 |  |
| Congo Democratic Republic | rural | 59.6 | 41.8 | 35.9 | 48.0 |  |
| Costa Rica | urban non-poor | 35.0 | 0.8 | 0.2 | 3.3 | **0.030** |
| Costa Rica | urban poor | 38.1 | 3.0 | 0.9 | 9.1 |  |
| Costa Rica | rural | 26.9 | 3.3 | 1.7 | 6.3 |  |
| Cote dIvoire | urban non-poor | 20.5 | 10.0 | 6.0 | 16.2 | **< 0.01** |
| Cote dIvoire | urban poor | 19.4 | 19.8 | 14.3 | 26.8 |  |
| Cote dIvoire | rural | 60.1 | 22.8 | 19.7 | 26.3 |  |
| Cuba | urban non-poor | 33.6 | 0.8 | 0.3 | 2.4 | 0.105 |
| Cuba | urban poor | 28.5 | 4.8 | 2.2 | 10.4 |  |
| Cuba | rural | 37.8 | 2.5 | 0.6 | 9.4 |  |
| Dominican Republic | urban non-poor | 40.7 | 6.7 | 4.3 | 10.4 | 0.368 |
| Dominican Republic | urban poor | 35.5 | 8.5 | 6.0 | 11.7 |  |
| Dominican Republic | rural | 23.8 | 9.8 | 7.1 | 13.4 |  |
| Egypt | urban non-poor | 17.9 | 0.3 | 0.0 | 1.5 | 0.216 |
| Egypt | urban poor | 12.2 | 0.2 | 0.1 | 1.0 |  |
| Egypt | rural | 69.9 | 0.7 | 0.4 | 1.4 |  |
| El Salvador | urban non-poor | 29.6 | 2.1 | 0.5 | 7.8 | 0.143 |
| El Salvador | urban poor | 29.0 | 0.3 | 0.1 | 1.1 |  |
| El Salvador | rural | 41.4 | 1.0 | 0.5 | 2.1 |  |
| Eswatini | urban non-poor | 13.1 | 2.9 | 0.6 | 11.9 | 0.806 |
| Eswatini | urban poor | 9.1 | 5.9 | 1.4 | 21.5 |  |
| Eswatini | rural | 77.8 | 3.2 | 1.8 | 5.6 |  |
| Ethiopia | urban non-poor | 21.1 | 4.7 | 1.7 | 12.4 | **< 0.01** |
| Ethiopia | urban poor | 9.4 | 21.8 | 10.1 | 41.1 |  |
| Ethiopia | rural | 69.5 | 29.7 | 23.8 | 36.3 |  |
| Fiji | urban non-poor | 29.0 | 2.2 | 0.5 | 8.6 | 0.723 |
| Fiji | urban poor | 24.2 | 4.2 | 1.6 | 10.7 |  |
| Fiji | rural | 46.7 | 3.6 | 1.7 | 7.3 |  |
| Gabon | urban non-poor | 44.1 | 9.1 | 5.1 | 15.4 | **0.022** |
| Gabon | urban poor | 39.1 | 11.5 | 7.9 | 16.3 |  |
| Gabon | rural | 16.9 | 18.3 | 13.7 | 24.1 |  |
| Gambia | urban non-poor | 38.6 | 2.4 | 1.0 | 5.8 | 0.271 |
| Gambia | urban poor | 27.7 | 1.7 | 0.6 | 4.5 |  |
| Gambia | rural | 33.8 | 0.8 | 0.3 | 1.9 |  |
| Ghana | urban non-poor | 27.3 | 3.5 | 1.8 | 6.7 | 0.899 |
| Ghana | urban poor | 16.9 | 4.1 | 2.0 | 8.2 |  |
| Ghana | rural | 55.9 | 4.3 | 2.5 | 7.0 |  |
| Guatemala | urban non-poor | 17.7 | 1.0 | 0.3 | 3.4 | 0.065 |
| Guatemala | urban poor | 17.9 | 2.3 | 1.1 | 4.7 |  |
| Guatemala | rural | 64.4 | 2.9 | 2.1 | 4.1 |  |
| Guinea | urban non-poor | 18.4 | 17.9 | 12.7 | 24.7 | **< 0.01** |
| Guinea | urban poor | 11.6 | 26.9 | 19.5 | 35.9 |  |
| Guinea | rural | 69.9 | 44.7 | 40.1 | 49.4 |  |
| Guinea Bissau | urban non-poor | 14.5 | 8.4 | 4.3 | 15.5 | 0.629 |
| Guinea Bissau | urban poor | 11.7 | 9.5 | 5.0 | 17.5 |  |
| Guinea Bissau | rural | 73.9 | 6.4 | 4.4 | 9.1 |  |
| Guyana | urban non-poor | 11.3 | 5.1 | 1.8 | 13.5 | 0.369 |
| Guyana | urban poor | 8.5 | 13.5 | 5.8 | 28.3 |  |
| Guyana | rural | 80.2 | 8.7 | 5.7 | 13.1 |  |
| Haiti | urban non-poor | 16.6 | 7.8 | 3.8 | 15.6 | **0.015** |
| Haiti | urban poor | 17.9 | 14.8 | 9.9 | 21.7 |  |
| Haiti | rural | 65.5 | 19.1 | 14.8 | 24.3 |  |
| Honduras | urban non-poor | 24.0 | 3.3 | 1.6 | 6.7 | 0.832 |
| Honduras | urban poor | 18.3 | 2.9 | 1.3 | 6.4 |  |
| Honduras | rural | 57.7 | 3.7 | 2.5 | 5.6 |  |
| India | urban non-poor | 14.6 | 5.9 | 5.0 | 6.9 | **< 0.01** |
| India | urban poor | 12.3 | 9.4 | 7.7 | 11.4 |  |
| India | rural | 73.1 | 5.9 | 5.6 | 6.3 |  |
| Indonesia | urban non-poor | 29.6 | 7.0 | 5.2 | 9.3 | **< 0.01** |
| Indonesia | urban poor | 19.3 | 11.5 | 9.0 | 14.6 |  |
| Indonesia | rural | 51.2 | 13.4 | 11.3 | 15.9 |  |
| Iraq | urban non-poor | 38.5 | 9.6 | 7.1 | 12.8 | **< 0.01** |
| Iraq | urban poor | 30.1 | 14.7 | 11.3 | 18.8 |  |
| Iraq | rural | 31.3 | 16.6 | 13.2 | 20.7 |  |
| Jamaica | urban non-poor | 32.0 | 2.2 | 0.7 | 6.8 | 0.390 |
| Jamaica | urban poor | 23.3 | 8.6 | 2.2 | 28.1 |  |
| Jamaica | rural | 44.7 | 4.8 | 2.2 | 10.2 |  |
| Jordan | urban non-poor | 42.6 | 6.6 | 3.9 | 11.0 | 0.141 |
| Jordan | urban poor | 45.6 | 8.9 | 6.3 | 12.3 |  |
| Jordan | rural | 11.8 | 4.8 | 2.7 | 8.3 |  |
| Kazakhstan | urban non-poor | 27.2 | 7.5 | 4.5 | 12.4 | **0.045** |
| Kazakhstan | urban poor | 21.0 | 4.7 | 2.7 | 8.0 |  |
| Kazakhstan | rural | 51.7 | 2.6 | 1.5 | 4.4 |  |
| Kenya | urban non-poor | 22.1 | 1.9 | 0.7 | 5.2 | 0.570 |
| Kenya | urban poor | 13.1 | 2.0 | 1.0 | 4.0 |  |
| Kenya | rural | 64.8 | 2.7 | 2.0 | 3.7 |  |
| Kiribati | urban non-poor | 31.2 | 38.8 | 30.2 | 48.2 | 0.349 |
| Kiribati | urban poor | 23.9 | 46.8 | 36.7 | 57.3 |  |
| Kiribati | rural | 44.8 | 37.5 | 29.9 | 45.7 |  |
| Kosovo | urban non-poor | 22.2 | 2.6 | 0.6 | 9.8 | 0.640 |
| Kosovo | urban poor | 14.9 | 5.7 | 2.0 | 14.8 |  |
| Kosovo | rural | 62.9 | 4.0 | 1.8 | 8.6 |  |
| Kyrgyzstan | urban non-poor | 17.6 | 17.5 | 10.7 | 27.4 | 0.069 |
| Kyrgyzstan | urban poor | 15.2 | 11.3 | 4.6 | 25.3 |  |
| Kyrgyzstan | rural | 67.2 | 6.8 | 4.3 | 10.4 |  |
| Lao | urban non-poor | 17.6 | 15.9 | 11.8 | 21.2 | **< 0.01** |
| Lao | urban poor | 12.1 | 31.2 | 24.7 | 38.6 |  |
| Lao | rural | 70.3 | 29.2 | 26.2 | 32.4 |  |
| Lesotho | urban non-poor | 18.0 | 8.9 | 4.4 | 17.2 | 0.668 |
| Lesotho | urban poor | 15.0 | 6.2 | 2.8 | 13.3 |  |
| Lesotho | rural | 67.0 | 8.8 | 6.1 | 12.6 |  |
| Liberia | urban non-poor | 28.4 | 6.7 | 3.1 | 13.9 | 0.375 |
| Liberia | urban poor | 23.2 | 6.9 | 2.9 | 15.7 |  |
| Liberia | rural | 48.3 | 10.4 | 8.0 | 13.4 |  |
| Madagascar | urban non-poor | 8.1 | 6.7 | 4.0 | 11.3 | **< 0.01** |
| Madagascar | urban poor | 7.9 | 20.5 | 13.2 | 30.5 |  |
| Madagascar | rural | 84.0 | 23.3 | 20.7 | 26.2 |  |
| Malawi | urban non-poor | 6.9 | 3.5 | 1.5 | 8.1 | 0.771 |
| Malawi | urban poor | 6.5 | 5.4 | 2.3 | 12.0 |  |
| Malawi | rural | 86.5 | 4.6 | 3.6 | 5.8 |  |
| Maldives | urban non-poor | 20.7 | 13.5 | 5.7 | 28.7 | **< 0.01** |
| Maldives | urban poor | 12.4 | 0.0 |  |  |  |
| Maldives | rural | 66.9 | 9.6 | 7.3 | 12.6 |  |
| Mali | urban non-poor | 11.9 | 4.2 | 2.1 | 8.4 | **< 0.01** |
| Mali | urban poor | 9.1 | 12.7 | 8.0 | 19.6 |  |
| Mali | rural | 79.0 | 20.6 | 17.1 | 24.6 |  |
| Mauritania | urban non-poor | 25.6 | 9.0 | 5.4 | 14.7 | 0.244 |
| Mauritania | urban poor | 18.1 | 11.8 | 8.2 | 16.9 |  |
| Mauritania | rural | 56.3 | 13.7 | 10.9 | 17.1 |  |
| Mexico | urban non-poor | 35.5 | 7.4 | 4.0 | 13.2 | 0.321 |
| Mexico | urban poor | 37.6 | 9.8 | 6.1 | 15.4 |  |
| Mexico | rural | 26.9 | 5.5 | 3.0 | 9.9 |  |
| Mongolia | urban non-poor | 35.1 | 3.2 | 1.3 | 7.6 | 0.220 |
| Mongolia | urban poor | 32.2 | 1.7 | 0.7 | 4.0 |  |
| Mongolia | rural | 32.8 | 3.9 | 2.3 | 6.4 |  |
| Montenegro | urban non-poor | 35.9 | 3.9 | 1.2 | 12.5 | 0.731 |
| Montenegro | urban poor | 23.5 | 7.1 | 1.7 | 24.9 |  |
| Montenegro | rural | 40.6 | 6.1 | 2.4 | 14.6 |  |
| Mozambique | urban non-poor | 13.5 | 2.8 | 1.2 | 6.5 | **< 0.01** |
| Mozambique | urban poor | 11.9 | 6.7 | 2.6 | 16.1 |  |
| Mozambique | rural | 74.6 | 11.8 | 7.8 | 17.6 |  |
| Myanmar | urban non-poor | 14.1 | 1.9 | 0.3 | 11.6 | **< 0.01** |
| Myanmar | urban poor | 11.8 | 17.6 | 8.4 | 33.0 |  |
| Myanmar | rural | 74.1 | 14.6 | 10.9 | 19.2 |  |
| Namibia | urban non-poor | 23.8 | 13.0 | 7.8 | 21.0 | **< 0.01** |
| Namibia | urban poor | 25.9 | 9.0 | 5.4 | 14.8 |  |
| Namibia | rural | 50.4 | 3.8 | 2.4 | 6.0 |  |
| Nepal | urban non-poor | 36.6 | 7.0 | 4.5 | 10.7 | **< 0.01** |
| Nepal | urban poor | 28.3 | 16.8 | 12.3 | 22.4 |  |
| Nepal | rural | 35.1 | 9.2 | 6.7 | 12.4 |  |
| Niger | urban non-poor | 8.8 | 19.5 | 12.9 | 28.2 | 0.837 |
| Niger | urban poor | 4.5 | 17.0 | 9.5 | 28.6 |  |
| Niger | rural | 86.7 | 19.5 | 15.5 | 24.3 |  |
| Nigeria | urban non-poor | 23.3 | 10.9 | 8.7 | 13.5 | **< 0.01** |
| Nigeria | urban poor | 16.2 | 30.3 | 25.8 | 35.1 |  |
| Nigeria | rural | 60.5 | 45.0 | 42.5 | 47.5 |  |
| North Macedonia | urban non-poor | 41.2 | 3.1 | 0.7 | 12.5 | 0.292 |
| North Macedonia | urban poor | 17.7 | 13.4 | 4.7 | 32.5 |  |
| North Macedonia | rural | 41.1 | 1.2 | 0.3 | 4.8 |  |
| Pakistan | urban non-poor | 20.4 | 4.3 | 2.5 | 7.3 | **< 0.01** |
| Pakistan | urban poor | 14.0 | 13.2 | 8.6 | 19.6 |  |
| Pakistan | rural | 65.7 | 16.6 | 13.3 | 20.7 |  |
| Panama | urban non-poor | 26.3 | 6.9 | 2.6 | 16.9 | 0.387 |
| Panama | urban poor | 31.4 | 10.6 | 6.2 | 17.6 |  |
| Panama | rural | 42.3 | 6.2 | 4.1 | 9.2 |  |
| Papua New Guinea | urban non-poor | 6.6 | 13.8 | 9.6 | 19.5 | **< 0.01** |
| Papua New Guinea | urban poor | 5.6 | 22.6 | 11.0 | 40.7 |  |
| Papua New Guinea | rural | 87.8 | 38.7 | 34.3 | 43.2 |  |
| Paraguay | urban non-poor | 31.9 | 6.6 | 4.3 | 10.0 | **< 0.01** |
| Paraguay | urban poor | 30.2 | 2.1 | 0.9 | 4.8 |  |
| Paraguay | rural | 37.9 | 6.5 | 4.4 | 9.7 |  |
| Peru | urban non-poor | 40.8 | 7.0 | 5.4 | 9.0 | 0.397 |
| Peru | urban poor | 36.0 | 6.8 | 5.4 | 8.5 |  |
| Peru | rural | 23.2 | 8.5 | 6.7 | 10.7 |  |
| Philippines | urban non-poor | 23.6 | 8.4 | 5.3 | 13.1 | **0.015** |
| Philippines | urban poor | 22.2 | 14.0 | 9.8 | 19.5 |  |
| Philippines | rural | 54.2 | 15.3 | 12.9 | 18.1 |  |
| Rwanda | urban non-poor | 8.5 | 2.2 | 0.7 | 6.6 | 0.075 |
| Rwanda | urban poor | 8.0 | 0.0 |  |  |  |
| Rwanda | rural | 83.5 | 0.2 | 0.1 | 0.8 |  |
| Samoa | urban non-poor | 8.6 | 21.5 | 12.3 | 34.9 | 0.128 |
| Samoa | urban poor | 7.4 | 21.6 | 14.1 | 31.5 |  |
| Samoa | rural | 84.0 | 31.0 | 25.8 | 36.9 |  |
| Sao Tome and Principe | urban non-poor | 36.7 | 1.3 | 0.2 | 8.7 | 0.621 |
| Sao Tome and Principe | urban poor | 25.4 | 2.2 | 0.5 | 8.7 |  |
| Sao Tome and Principe | rural | 37.9 | 3.3 | 1.3 | 8.1 |  |
| Senegal | urban non-poor | 22.3 | 0.9 | 0.2 | 3.4 | **< 0.01** |
| Senegal | urban poor | 15.6 | 3.1 | 0.7 | 13.2 |  |
| Senegal | rural | 62.1 | 5.0 | 3.4 | 7.5 |  |
| Serbia | urban non-poor | 43.7 | 2.3 | 0.6 | 9.4 | **0.018** |
| Serbia | urban poor | 14.5 | 0.0 |  |  |  |
| Serbia | rural | 41.8 | 6.0 | 2.9 | 12.2 |  |
| Sierra Leone | urban non-poor | 20.4 | 3.8 | 1.8 | 7.9 | 0.411 |
| Sierra Leone | urban poor | 13.8 | 4.8 | 2.7 | 8.5 |  |
| Sierra Leone | rural | 65.8 | 6.0 | 4.5 | 7.9 |  |
| South Africa | urban non-poor | 29.5 | 8.2 | 3.9 | 16.6 | 0.624 |
| South Africa | urban poor | 32.0 | 11.0 | 6.1 | 18.8 |  |
| South Africa | rural | 38.6 | 7.6 | 5.1 | 11.0 |  |
| South Sudan | urban non-poor | 14.7 | 59.6 | 51.5 | 67.3 | **< 0.01** |
| South Sudan | urban poor | 10.6 | 73.3 | 65.1 | 80.1 |  |
| South Sudan | rural | 74.7 | 75.2 | 71.0 | 78.9 |  |
| State of Palestine | urban non-poor | 50.2 | 7.0 | 4.9 | 9.9 | **< 0.01** |
| State of Palestine | urban poor | 34.1 | 0.3 | 0.1 | 2.0 |  |
| State of Palestine | rural | 15.7 | 5.6 | 3.3 | 9.3 |  |
| Sudan | urban non-poor | 15.2 | 6.3 | 4.1 | 9.7 | **< 0.01** |
| Sudan | urban poor | 12.0 | 13.9 | 9.5 | 19.9 |  |
| Sudan | rural | 72.8 | 19.4 | 16.0 | 23.4 |  |
| Suriname | urban non-poor | 33.8 | 19.7 | 12.1 | 30.4 | 0.897 |
| Suriname | urban poor | 33.3 | 20.8 | 14.7 | 28.7 |  |
| Suriname | rural | 32.9 | 18.6 | 13.3 | 25.5 |  |
| Tajikistan | urban non-poor | 10.6 | 10.5 | 7.2 | 15.1 | **0.043** |
| Tajikistan | urban poor | 8.7 | 13.6 | 7.6 | 23.2 |  |
| Tajikistan | rural | 80.7 | 6.6 | 5.0 | 8.6 |  |
| Tanzania | urban non-poor | 16.3 | 1.3 | 0.4 | 4.1 | **< 0.01** |
| Tanzania | urban poor | 12.4 | 0.6 | 0.1 | 2.6 |  |
| Tanzania | rural | 71.4 | 3.9 | 2.8 | 5.4 |  |
| Thailand | urban non-poor | 18.7 | 2.4 | 0.9 | 6.2 | 0.429 |
| Thailand | urban poor | 16.6 | 8.2 | 2.6 | 23.2 |  |
| Thailand | rural | 64.6 | 2.0 | 1.4 | 3.0 |  |
| Timor Leste | urban non-poor | 14.7 | 9.7 | 5.1 | 17.5 | **< 0.01** |
| Timor Leste | urban poor | 13.1 | 17.2 | 11.6 | 24.7 |  |
| Timor Leste | rural | 72.2 | 24.8 | 21.6 | 28.4 |  |
| Togo | urban non-poor | 24.5 | 6.5 | 3.4 | 12.1 | 0.313 |
| Togo | urban poor | 15.2 | 7.5 | 3.3 | 16.3 |  |
| Togo | rural | 60.3 | 10.6 | 7.7 | 14.6 |  |
| Tonga | urban non-poor | 12.1 | 5.7 | 1.4 | 20.1 | 0.136 |
| Tonga | urban poor | 10.3 | 0.0 |  |  |  |
| Tonga | rural | 77.6 | 3.8 | 1.3 | 10.6 |  |
| Tunisia | urban non-poor | 40.6 | 8.7 | 5.6 | 13.5 | **< 0.01** |
| Tunisia | urban poor | 20.6 | 3.3 | 1.4 | 7.5 |  |
| Tunisia | rural | 38.8 | 1.0 | 0.3 | 3.0 |  |
| Türkiye | urban non-poor | 39.3 | 3.0 | 1.2 | 7.2 | 0.131 |
| Türkiye | urban poor | 40.2 | 8.6 | 5.0 | 14.5 |  |
| Türkiye | rural | 20.5 | 4.7 | 2.2 | 9.5 |  |
| Turkmenistan | urban non-poor | 20.1 | 2.2 | 0.8 | 5.7 | 0.051 |
| Turkmenistan | urban poor | 15.4 | 1.5 | 0.3 | 5.9 |  |
| Turkmenistan | rural | 64.5 | 0.0 |  |  |  |
| Tuvalu | urban non-poor | 34.9 | 0.0 |  |  | 0.377 |
| Tuvalu | urban poor | 24.2 | 4.0 | 0.6 | 22.7 |  |
| Tuvalu | rural | 40.9 | 1.9 | 0.2 | 13.5 |  |
| Uganda | urban non-poor | 15.3 | 5.6 | 3.0 | 10.4 | 0.949 |
| Uganda | urban poor | 8.2 | 4.9 | 2.1 | 11.0 |  |
| Uganda | rural | 76.6 | 5.0 | 4.0 | 6.3 |  |
| Ukraine | urban non-poor | 45.1 | 16.3 | 10.8 | 23.8 | 0.778 |
| Ukraine | urban poor | 26.9 | 13.4 | 7.6 | 22.6 |  |
| Ukraine | rural | 28.0 | 16.7 | 11.2 | 24.3 |  |
| Vietnam | urban non-poor | 20.6 | 4.2 | 1.3 | 12.4 | 0.371 |
| Vietnam | urban poor | 10.7 | 10.6 | 4.7 | 22.1 |  |
| Vietnam | rural | 68.7 | 4.2 | 2.7 | 6.5 |  |
| Yemen | urban non-poor | 15.3 | 8.2 | 5.7 | 11.6 | **< 0.01** |
| Yemen | urban poor | 11.2 | 22.6 | 16.7 | 29.8 |  |
| Yemen | rural | 73.5 | 26.7 | 23.9 | 29.7 |  |
| Zambia | urban non-poor | 19.6 | 1.3 | 0.4 | 4.0 | 0.437 |
| Zambia | urban poor | 15.6 | 1.8 | 0.6 | 5.3 |  |
| Zambia | rural | 64.8 | 2.4 | 1.6 | 3.7 |  |
| Zimbabwe | urban non-poor | 15.3 | 1.8 | 0.7 | 4.8 | **0.013** |
| Zimbabwe | urban poor | 13.4 | 3.3 | 1.4 | 7.8 |  |
| Zimbabwe | rural | 71.3 | 6.7 | 4.6 | 9.7 |  |

* Wald test for difference between groups. Bold font stands for p value < 0.05.
